# Attitudes and behaviour regarding dose reduction of biologics for psoriasis: a survey among dermatologists worldwide

**DOI:** 10.1007/s00403-021-02273-4

**Published:** 2021-08-31

**Authors:** M. E. van Muijen, L. S. van der Schoot, J. M. P. A. van den Reek, E. M. G. J. de Jong

**Affiliations:** 1 Department of Dermatology, Radboud University Medical Center (Radboudumc), Nijmegen, The Netherlands; 2 Radboud Institute for Health Sciences (RIHS), Radboud University Medical Center (Radboudumc), Nijmegen, The Netherlands; 3 Radboud University, Nijmegen, The Netherlands

**Keywords:** Psoriasis, Biologics, Dose reduction, Survey, Dermatologists

## Abstract

Dose reduction (DR) of biologics, where possible, seems promising for more efficient use of expensive biologics. For implementation of DR strategies, it is essential to get insight in factors that influence implementation. The objective of this study was to evaluate the attitudes and behaviour regarding dose reduction of biologic therapies for psoriasis among psoriasis expert dermatologists worldwide. A 27-question e-survey was sent through the International Psoriasis Council (IPC) to its 114 dermatologist councilors worldwide. The survey assessed demographics, general and DR prescription behaviour, and motivations for and barriers against application of DR. Of 57 respondents, 53 respondents who prescribed biologics were included for analysis. Thirty-seven (69.8%) applied DR (i.e., ‘DR dermatologists’), and 16 (30.2%) did not (i.e., ‘Non-DR dermatologists’). DR strategies varied among respondents. Regarding criteria for starting DR, differences were reported in required treatment duration, and interpretation and duration of stable low disease activity. In addition, the prolongation of intervals between injections varied between respondents. For most ‘DR dermatologists’ (*n* = 32/37, 86.5%), cost savings were one of the main reasons to apply DR. Fifteen out of 16 ‘Non-DR dermatologists’ (94%) did not apply DR due to lack of scientific evidence. In conclusion, DR of biologics for psoriasis is part of clinical practice in psoriasis experts globally. Barriers for applying DR included lack of evidence or guidelines, and uncertainty on DR effects and risks. Although growing evidence shows DR feasibility, future studies are needed to accumulate and broaden evidence, along with development of (inter)national guidelines on DR strategies.

**Supplementary Information:**

The online version contains supplementary material available at 10.1007/s00403-021-02273-4.

## Introduction

Biologics have expanded treatment options for psoriasis in the last decades. These drugs reduce skin symptoms and improve quality of life in psoriasis patients [5]. Besides their effectiveness, biologics are expensive and impose a high burden on national health care expenditures. In addition, it is important to strive for the lowest effective dose, to reduce the risk of side effects. Therefore, personalized and efficient use of biologics is warranted. Biologics are often prescribed in a fixed, registered dose, whereas patients with a good response might not need this standard dose.

Dose reduction (DR) of biologics (also referred to as ‘dose tapering’), seems, therefore, a promising way for more efficient and safer use of biologics. By striving for the lowest effective dose, overtreatment can be prevented and healthcare costs can be reduced. To date, studies on biologic DR report different strategies, but DR seems feasible and safe in a substantial part of patients with low disease activity [2–4, 9, 16, 18, 20, 21]. However, studies to date mostly focussed on TNFa-inhibitors and ustekinumab, and information on the newer biologics is sparse [13].

For further implementation of DR strategies worldwide, it is essential to get more insight in factors which influence implementation [7]. Possible barriers which might prevent application of DR are for example knowledge and attitudes of the involved patients and dermatologists. In addition, local organization of healthcare, and availability of expertise and resources, should be taken into account. While previous studies mainly focused on clinical DR outcomes in local settings, little is known about the current daily practice and attitude towards DR of dermatologists worldwide. Therefore, in 2020, an international survey was sent via the International Psoriasis Council to its dermatologist councilors worldwide, with the aim to evaluate their thoughts and behaviour regarding biologic DR in psoriasis patients.

## Materials and methods

### Target population and survey methodology

A questionnaire was developed based on a previous questionnaire sent to Dutch dermatologists [22]. All questions were reviewed by the International Psoriasis Council (IPC) chief executive officer for face validity. The survey was designed using online questionnaire and data repository software Qualtrics (XM 2020, Provo, UT, USA). The target population consisted of dermatologists worldwide, affiliated with the IPC as councilor, and who prescribed biological therapies for psoriasis patients. The 27-question survey and an introduction e-mail were sent electronically via the IPC on 28 July 2020 to all IPC councilors (*n* = 114). To maximize response rates, a reminder was sent after 10 weeks. The online survey was closed on 30 October 2020. All participant responses were anonymously collected using unique respondent identification numbers.

This study was reviewed by the ethics committee of the region of Arnhem-Nijmegen and Radboud University Medical Center and was deemed to not fall within the remit of the Medical Research Involving Human Subjects Act (2021-13093), as we did not collect any personal data. Therefore, informed consent from participants was not mandatory. Consent to participate was assumed in case of completion of the e-survey. The study was conducted in accordance with the ethical principles of the Declaration of Helsinki.

### Survey content

Dose reduction was defined in the survey as ‘the application of injection interval prolongation’ and/or’decreasing the absolute dose in number of milligrams per administration’. The survey addressed demographics (country and place of work), prescription behaviour of biologics for psoriasis in general (numbers of patients treated with biologics, clinical scores used for measuring disease activity), application of DR (attitudes towards DR, reasons for applying or not applying DR, DR regimen per biologic, conditions for applying DR, success rates of applied DR). At last, respondents were asked for barriers which might prevent them from application of DR. Both open answers and predefined answers were used. In case of predefined answers, there was an option to add comments. Questions regarding DR were only displayed to respondents who indicated that they applied DR. For the complete questionnaire, see Supplement S1.

### Analysis

Only completed surveys were included for analysis. Descriptive statistics were calculated to describe survey responses. As the number of respondents exposed to each question differed, results are presented as absolute numbers with percentages of respondents that were exposed to the question. All analyses were conducted in SPSS Statistics 25 (IBM, Armonk, NY, U.S.A).

## Results

A total of 57/114 surveys were completed, indicating a response rate of 50%. Four respondents were excluded from analyses as they did not prescribe biologics or biosimilars for psoriasis. Among 53 dermatologists (46.5%) that prescribed biologics or biosimilars for psoriasis, 35.8% were from Europe (Denmark [*n* = 5], Germany [*n* = 2], Italy [*n* = 3], Portugal [*n* = 2], Sweden [*n* = 1], Switzerland [*n* = 2], The Netherlands [*n* = 1], United Kingdom [*n* = 3]), 24.5% from South America (Argentina [*n* = 3], Brazil [*n* = 2], Chile [*n* = 3], Colombia [*n* = 5]), 17.0% from Asia (China [*n* = 2], Iran [*n* = 1], Israel [*n* = 1], Japan [*n* = 3], Malaysia [*n* = 1], Singapore [*n* = 1]), 15.0% from North America (Canada [*n* = 4], Guatemala [*n* = 1], USA [*n* = 3]), 5.7% from Africa (Egypt [*n* = 3]), and 1.9% from Australia (Australia [*n* = 1]). The majority (*n* = 33, 62.3%) was employed in an academic hospital. The majority of respondents (*n* = 27, 50.9%) estimated the total number of patients treated with biologics at their departments between 100 and 500. Ten respondents (18.9%) estimated this number as < 100, whereas 15 respondents (28.3%) estimated that this number was > 500, and 1 respondent did not know. Biosimilars were prescribed by 66.0% (*n* = 35). Dose reduction was applied by 37 dermatologists (69.8%) (further called ‘DR dermatologists’), and 16 dermatologists (30.2%) did not apply DR (‘Non-DR dermatologists’).

### *Prescription behaviour and monitoring of psoriasis disease activity (n* = *53)*

Ustekinumab and secukinumab were prescribed by the highest absolute number of respondents (*n* = 51, 96.2%), whereas brodalumab and tildrakizumab were prescribed least often (*n* = 24, 45.3%, and *n* = 14, 26.4%, respectively). Tools that were used for measurement of disease activity were PASI, Body Surface Area (BSA) and Physician Global Assessment (PGA) by, respectively, *n* = 46 (86.8%), *n* = 42 (79.2%), and *n* = 28 (52.8%) of the respondents (multiple answers possible). One dermatologist (1.9%) did not use a disease activity score in daily practice. Six respondents replied ‘other’, and described using the DLQI (*n* = 2), Visual Analogue Scale (VAS) itch (*n* = 1), photo documentation (*n* = 1), subjective impact (*n* = 1), or a ‘VAS score of the patient’ (*n* = 1).

### *DR eligibility criteria and regimens (n* = *37 ‘DR dermatologists’)*

Criteria for applying DR are presented in Table 1. Nine respondents (24.3%) would only consider DR if patients were free from psoriasis (PASI/BSA/PGA 0). Seventeen respondents (45.9%) indicated a ‘PASI ≤ 1 or ≤ 2, BSA ≤ 1 or ≤ 2, or PGA ≤ 1’ as criterium to initiate DR. Two respondents (5.4%) would consider DR in PASI ≤ 3, *n* = 1 (2.7%) in PASI ≤ 5, *n* = 2 (5.4%) in BSA ≤ 3, and *n* = 1 (2.7%) in BSA ≤ 5%. DR criteria were based solely on disease activity by 23 respondents (43.4%), whereas 14 respondents (37.8%) used a combination of disease activity measures, side effects and/or patient preferences. The majority of DR dermatologists (*n* = 24, 64.9%) would consider DR after at least 1 year of treatment duration. Fifteen respondents (40.5%) considered stable low disease activity for the duration of at least 1 year prior to initiation of DR to be necessary. Detailed responses to questions on DR eligibility criteria are presented in Supplementary Tables S1 and S2.

**Table 1:** Criteria for applying dose reduction (DR) in ‘DR dermatologists’ (*N* = 37)

Question	*N* (%)
Criteria for starting dose reduction^a^	
In case of side effects	7 (18.9%)
At patients’ request	10 (27%)
Other	2 (5.4%)
Max. disease activity score for which DR is considered^b^	
PASI 0 / BSA 0 / PGA 0	9 (24.3%)
PASI ≤ 1 or ≤ 2 / BSA ≤ 1 or ≤ 2 / PGA ≤ 1	17 (45.9%)
PASI ≤ 3 or ≤ 5 / BSA ≤ 3 or ≤ 5	6 (16.2%)
Estimation of disease activity (clear/almost clear)	4 (10.8%)
Other	1 (2.7%)
Treatment duration	
At least 3 months	1 (2.7%)
At least 6 months	7 (18.9%)
At least 9 months	0
At least 1 year	24 (64.9%)
At least 2 years^b^	1 (2.7%)
Independent of treatment duration	3 (8.1%)
Other	3 (8.1%)
Duration stable low disease activity	
At least 6 weeks	1 (2.7%)
At least 3 months	8 (21.6%)
At least 6 months	8 (21.6%)
At least 9 months	2 (5.4%)
At least 1 year	15 (40.5%)
At least 2 years^b^	1 (2.7%)
Independent of duration stable low disease activity	0
Other	2 (5.4%)
Change of outpatient visits^a^	
Additional outpatient visit	2 (5.4%)
Additional telephone call	3 (8.1%)
Additional e-consult^c^	1 (2.7%)
Prolongation time between visits	13 (35.1%)
No adaptation	1 (2.7%)
Only at patients’ request	1 (2.7%)
Individualized per patient	2 (5.4%)

*PASI* psoriasis area and severity index, *BSA* body surface area

Data are presented as *N* (%) of total respondents applying dose reduction (‘DR dermatologists’)

^a^More answers were possible

^b^1 answer per respondent

^c^Answered in comment section

**Fig. 1:**
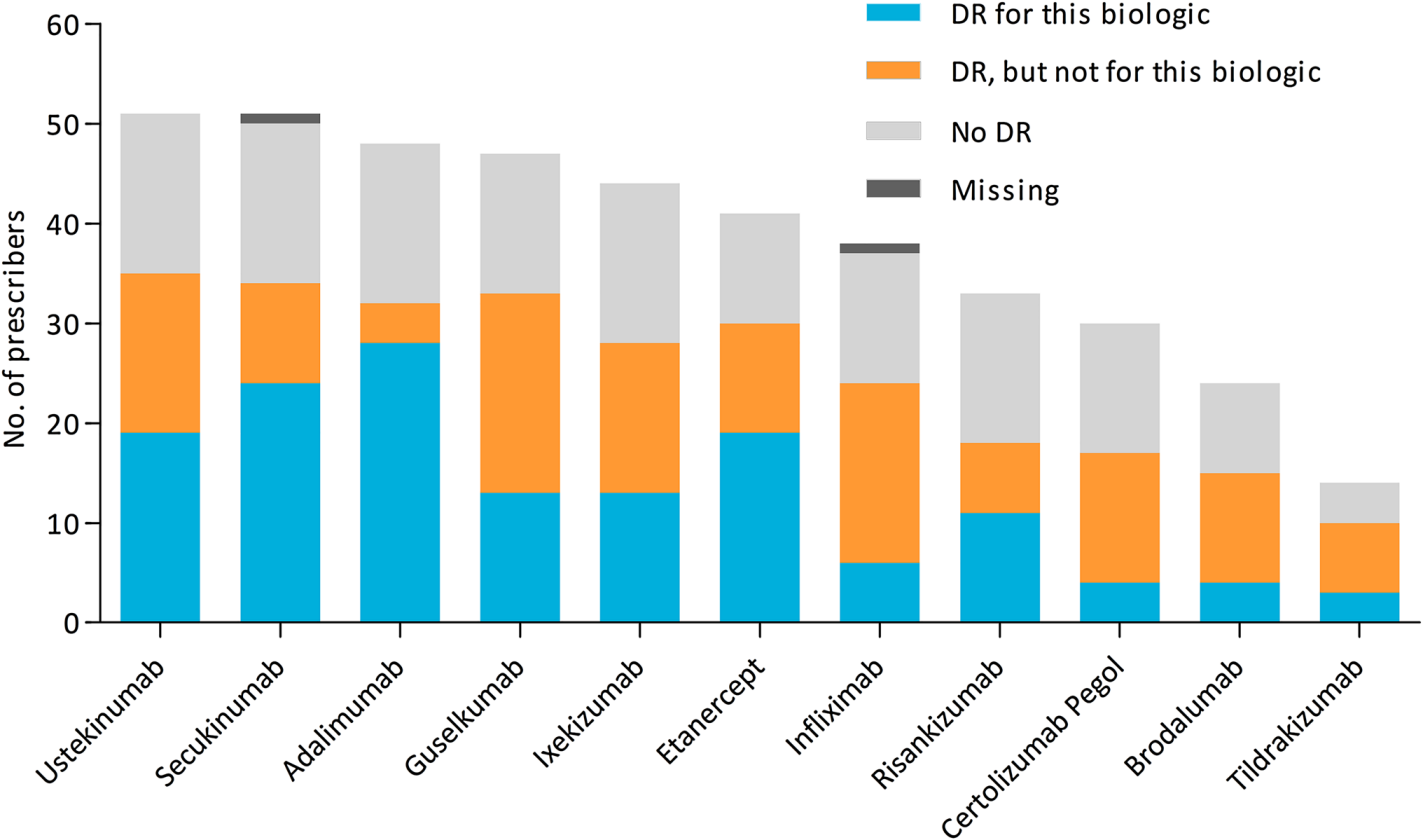
Dose reduction (DR) per biologic. Results are presented as absolute number of prescribers for each biologic, and the proportion of respondents that applied DR for each specific biologic. Respondents were first asked which biologics they prescribed. Subsequently they were asked to indicate whether they applied DR for the biologics they prescribed. Respondents who indicated to prescribe a specific biologic, but did not specify if they applied DR for that biologic, were accounted as missing. *DR* dose reduction

**Fig. 2:**
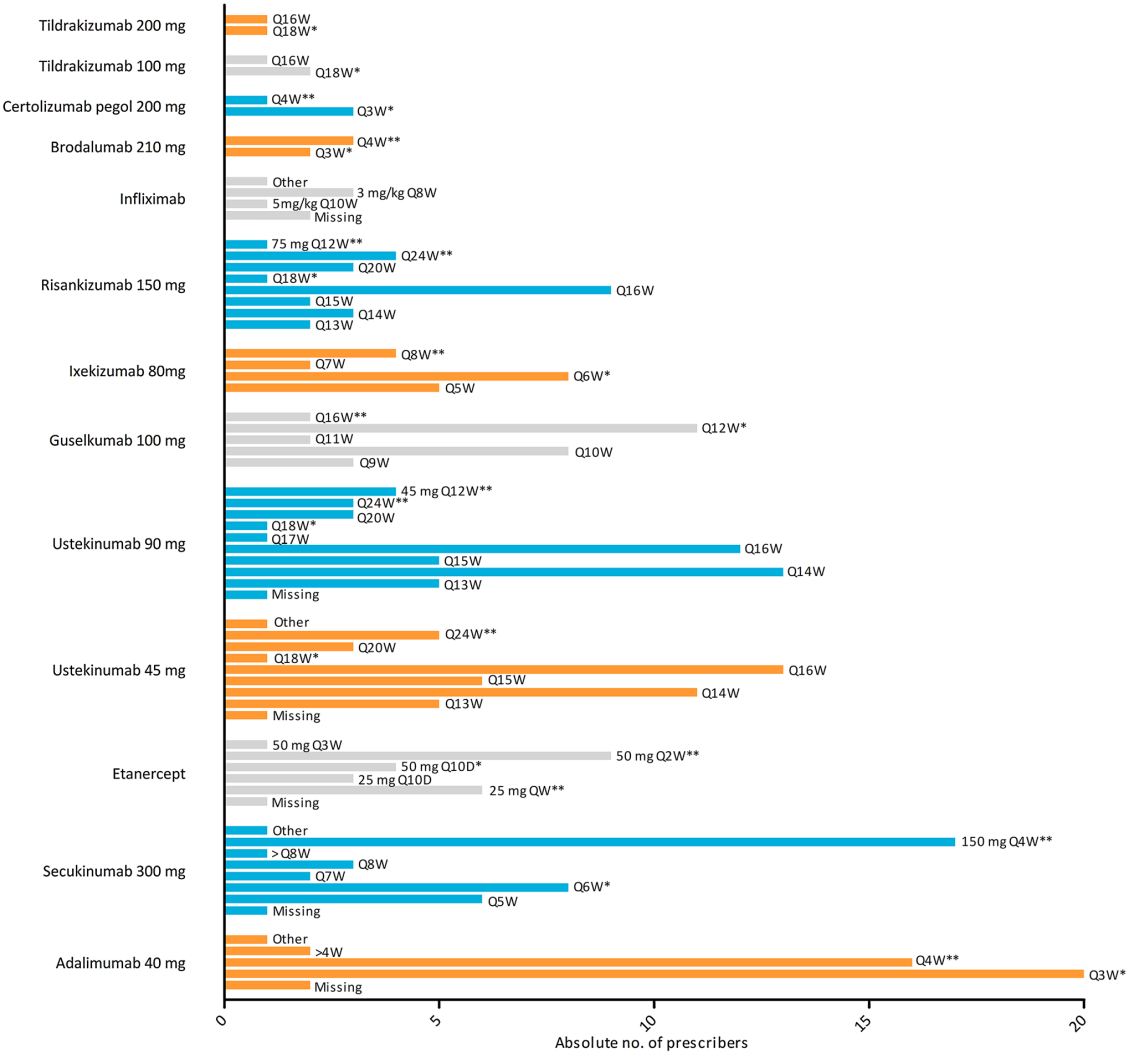
Dose reduction (DR) regimen per biologic. Results are presented as absolute number of prescribers for each biologic. Respondents were asked to indicate how they applied DR per biologic they prescribed. Multiple answers were possible. *33% reduction of the original dose, **50% reduction of the original dose**.** *QW* every week, *Q10D* every 10 days, *mg* milligram, *kg* kilogram

Figure 1 depicts the absolute number of prescribers for each biologic of the total number of respondents (*n* = 53), and the proportion of respondents that applied DR for each specific biologic. Dose reduction was applied by the largest number of ‘DR dermatologists’ for adalimumab (*n* = 28/37, 75.7%), secukinumab (*n* = 24/37, 64.9%), ustekinumab (*n* = 19/37, 51.4%), and etanercept (*n* = 19/37, 51.4%). Figure 2 displays the DR regimens applied by the absolute number of ‘DR dermatologists’ for each biologic separately. Two respondents indicated to reduce doses on individual basis without selecting predefined answer options. In general, smaller DR steps were used in biologics with long injection intervals, leading to relatively less reduction of the original dose, as opposed to biologics with shorter injection intervals.

### *Evaluation of patient eligibility, patient willingness and success rate of dose reduction (n* = *37 ‘DR dermatologists’)*

Table 2 provides a detailed overview of the estimations given by DR dermatologists on the percentage of patients in which they considered DR, patients’ willingness for DR, and success rates of DR. The majority (*n* = 29, 78.3%) estimated that their patients were frequently (‘often’ (*n* = 17, 45.9%) or ‘always’ (*n* = 12, 32.4%)) willing to start DR. There was a large variability in the evaluation of DR success, ranging from estimated success rates of < 20% (*n* = 3, 13.5%) to rates of > 80% (*n* = 4, 10.8%).

**Table 2:** Estimations by ‘DR dermatologists’ of patients on dose reduction (*N* = 37)

Question	*N* (%)
In how many percent of your patients do you consider dose reduction?^a^	
< 5%	12 (32.4%)
5–25%	16 (43.2%)
25–50%	7 (18.9%)
50–75%	1 (2.7%)
> 75%	0
How often do your patients agree to start dose reduction?	
Rarely	1 (2.7%)
Sometimes	7 (18.9%)
Often	17 (45.9%)
Always	12 (32.4%)
In what percentage of your patients that underwent dose reduction, the dose reduction strategy was successful?^b^	
< 20%	5 (13.5%)
20–40%	6 (16.2%)
40–60%	11 (29.7%)
60–80%	7 (18.9%)
> 80%	4 (10.8%)
I don’t know	3 (8.1%)

Data are presented as *N* (%) of total respondents applying dose reduction (i.e., ‘DR dermatologists’)

Missings: ^a^1, ^b^1

### *Reasons for re-increasing the dose (n* = *37 ‘DR dermatologists’)*

In addition, ‘DR dermatologists’ were inquired about their criteria to stop DR, and/or re-increase the biologic dose. Twenty-six (70.3%) respondents based this decision on disease activity parameters, *n* = 9 (24.3%) on a combination of disease activity and patients’ request, *n* = 1 (2.7%) decided to re-increase the dose solely on patients’ request, and *n* = 1 (2.7%) based this decision on ‘nothing particular’. Among ‘DR dermatologists’ that used disease activity scores (*n* = 26/37, 70.3%), *n* = 14 out of 26 (53.8%) would re-increase the dose in case of PASI or BSA ≥ 3. The maximum BSA at which a respondent would re-initiate treatment was BSA > 10% (*n* = 1/26, 3.8%). One respondent (*n* = 1/26, 3.8%) would re-increase the dose if total remission was lost (BSA > 0% or PGA > 0). Another respondent (*n* = 1/26, 3.8%) determined drug levels before re-increasing doses. Besides using disease activity measures, *n* = 13 respondents (35.1%) made a general estimation of disease activity, and would re-increase the dose in case of ‘moderate disease activity’. Detailed responses to the question on re-treatment criteria are provided in Supplementary Table S3.

### *Motivations and barriers for application of DR (n* = *37 ‘DR dermatologists’)*

Cost savings were one of the main reasons to apply DR (*n* = 32 out of *n* = 37 ‘DR dermatologists’, 86.5%). Fixed answer options ‘safety/less side effects’ and ‘at patients’ request’ were selected by *n* = 16 (43.2%) and *n* = 15 (40.5%) respondents, respectively (multiple answers possible). Two ‘DR dermatologists’ (5.4%) commented that patients should not be treated with more drugs than necessary. Twenty one (56.8%) ‘DR dermatologists’ would like to apply DR more often. Regarding reasons not to apply DR as much as they would like, *n* = 6 respondents felt that they were hampered by limited experience with DR, and *n* = 5 felt uncomfortable applying DR with biologics of the newer generations (IL-17/IL-23 inhibitors). Not having enough time (*n* = 3) or staff for support (*n* = 1), thinking the patient would not be interested (*n* = 1), and fearing financial consequences (*n* = 1) were other reasons not to apply DR more frequently. In addition, the risk of reduced effectiveness (*n* = 1) and lack of guidelines/scientific evidence (*n* = 3) were added as comments by respondents. To the question ‘Did you apply dose reduction more often during the COVID19 pandemic?’, *n* = 9 ‘DR dermatologists’ (24.3%) responded that they applied DR more often.

### *Barriers against DR (n* = *16 ‘Non-DR dermatologists’)*

Fifteen out of the 16 ‘Non-DR dermatologists’ (94%) did not apply DR due to the lack of scientific evidence on safety and efficacy of DR. However, *n* = 10 (62.5%) ‘Non-DR dermatologists’ indicated that they would consider DR if scientific evidence was available, and *n* = 6 (37.5%) would ‘maybe’ consider DR in that case. Apart from the lack of scientific evidence, frequently reported reasons not to apply DR were potential risk of psoriasis exacerbation (*n* = 9, 56.3%), fear of antibody formation (*n* = 7, 43.8%), loss of effectiveness (*n* = 7, 43.8%), and having the opinion that the costs of biologicals should be decreased instead of physicians applying DR (*n* = 7, 43.8%) (multiple answers possible).

### *Dermatologists’ attitudes towards DR (n* = *53, all respondents)*

Thirty-five respondents (66.0%) reported a positive attitude towards DR of biologics for psoriasis. Five respondents (9.4%) had a negative attitude towards DR, of which *n* = 4 actually applied DR themselves. Thirteen respondents (24.5%) described their attitude towards DR as neutral. Respondents were asked if they felt the necessity for a guideline on DR of biologics. In total, *n* = 33 (62.3%) indicated that they felt the necessity for a guideline on biologic DR, of which *n* = 32 indicated that scientific background information should be covered in such a guideline. Nine respondents (17.0%) selected the answer option ‘other’, of which *n* = 6 stated that more clinical trials on DR should be conducted prior to the development of a guideline. Ten respondents (18.9%) did not feel the necessity for a guideline.

## Discussion

Results of this survey among dermatologist councilors of the International Psoriasis Council showed that DR is applied in the treatment of psoriasis patients. Of 53 respondents, the majority (70%) applied DR, most frequently for the biologics adalimumab, etanercept, ustekinumab and secukinumab. Main reasons for application of DR were cost savings, safety, and patients’ request. Barriers against DR in dermatologists that already applied DR were limited experience with DR, limited experience with the newer biologics in general, not having enough time or support, risk of reduced effectiveness, and lack of guidelines or scientific evidence. Among dermatologists who did not apply DR, barriers were the lack of scientific evidence, potential risk of flares, fear for antibody formation, loss of efficacy, and the opinion that costs should be decreased by the pharmaceutical companies.

The used approaches differed among respondents. Globally, a more conservative approach was used in biologics with long injection intervals, leading to smaller DR steps, and, therefore, relatively less reduction of the original dose, as opposed to biologics with shorter injection intervals. The criteria for starting DR also varied among respondents. Most respondents required the patient to have stable low disease activity for at least 1 year or 6 months, but the definition of low disease activity varied among respondents. Almost half of the respondents (45.9%) would only consider DR if patients were free from psoriasis, whereas 6 respondents would still consider DR in case of PASI or BSA ≥ 3. This might be due to international differences in defined treatment goals and used outcomes [1, 6, 11, 14]. In defining criteria to initiate DR, or criteria for re-treatment in case of loss of response, various disease activity measures were used (PASI, BSA, PGA). Furthermore, some respondents would initiate DR based on a general estimation of the disease severity, making it difficult to draw general conclusions. Creating more uniform criteria to start and discontinue DR would facilitate further development of DR strategies.

Among barriers against DR were lack of guidelines and scientific evidence, and fear of disease flare. Currently, the option of biologic DR is only mentioned in a few guidelines [8, 17]. However, several studies have reported on the effects of DR in biologic therapies for psoriasis. Regarding the first generation biologics, several observational studies showed that DR of adalimumab, etanercept and ustekinumab is possible and safe in patients with low disease activity without losing disease control [3, 4, 9, 15, 16, 19–21]. In addition, a randomized controlled trial showed non-inferiority regarding quality of life but not regarding disease activity, although DR of adalimumab, etanercept, and ustekinumab was possible in 53% of patients, without safety concerns [2]. The development of anti-drug antibodies of ustekinumab did not differ between patients using a reduced dose versus the normal dose [4]. We recently conducted a scoping review on biologic DR in psoriasis, where we reported that for the newer IL-17 and IL-23 inhibitors, literature on DR was scarce [13]. Furthermore, a uniform DR strategy has not been described yet. However, most studies described a minimal treatment duration or stable low disease activity of 6–12 months, which is in line with the results of our survey. In most studies in the review, the biologic dose was gradually reduced in fixed steps, leading to 33% and 50% reduction of the original dose. In the present survey, DR steps differed between biologics and did not exceed 50% reduction for most biologics (Fig. 2). Regaining treatment response after relapse due to DR was achieved in most patients after re-treatment [2, 9, 16], although the number of studies on this topic were limited [13].

A strength of this study is the inclusion of dermatologists worldwide. To our knowledge this study is a first evaluation of attitudes in an international group of experts, specifically regarding biologic DR in psoriasis. Similar to the results of this survey, a national survey among Dutch dermatologists showed that DR was already applied in daily practice and also DR strategies differed [22]. Motivations for applying DR were comparable. However, barriers to applying DR in Dutch dermatologists were the belief that patients would not want to reduce their doses, forgetting to discuss the option of DR, or insufficient time for application of DR. Among respondents who did not apply DR at all, reasons were low experience with prescription of biologics in general or not knowing how to reduce the dose. Together with local differences in organization of healthcare, availability of resources, and internationally different treatment goals, these differences in experiences emphasize the need for local, tailored strategies and availability of consensus documents or guidelines. As a result of the COVID19 pandemic, some dermatologists stated that they applied DR more often. However, the effects of biologic therapies on susceptibility of COVID19 and COVID19 outcomes have not been fully elucidated, as well as the question if biologics should be interrupted [10, 12]. These questions add to reasons for further development of biologic DR strategies.

The main limitation of this study is the small sample size. For further validation of our results and for identifying global differences, replication in larger cohorts is needed. In addition, more structured methods that allow for consensus would be of value as well in future studies. By sending the survey through the International Psoriasis Council, there is a potential selection bias towards dermatologists with an interest in biological treatment, limiting the generalizability of our results.

In conclusion, the results of this worldwide survey among dermatologists show that 70% of responding psoriasis experts apply DR of biologics for psoriasis in clinical practice. However, respondents reported a large variety in used strategies regarding initiation and execution of DR. Dose reduction was applied less often in the more recently introduced biologics. Main motivations for applying DR were cost savings and improving safety. Among barriers against DR were the paucity of evidence or guidelines, and uncertainty on DR effect and risk of disease flares. Although growing evidence shows DR feasibility, future studies are needed for the development of local, tailored DR strategies and (inter)national guidelines.

## Supplementary Information

Below is the link to the electronic supplementary material.

Supplementary file 1 (PDF 409 KB)

## Data Availability

Upon request.

## References

[1] Armstrong AW, Siegel MP, Bagel J, Boh EE, Buell M, Cooper KD, Callis Duffin K, Eichenfield LF, Garg A, Gelfand JM, Gottlieb AB, Koo JY, Korman NJ, Krueger GG, Lebwohl MG, Leonardi CL, Mandelin AM, Menter MA, Merola JF, Pariser DM, Prussick RB, Ryan C, Shah KN, Weinberg JM, Williams MO, Wu JJ, Yamauchi PS, Van Voorhees AS (2017). From the Medical Board of the National Psoriasis Foundation: treatment targets for plaque psoriasis. J Am Acad Dermatol.

[2] Atalay S, van den Reek J, den Broeder AA, van Vugt LJ, Otero ME, Njoo MD, Mommers JM, Ossenkoppele PM, Koetsier MI, Berends MA, van de Kerkhof PCM, Groenewoud HMM, Kievit W, de Jong E (2020). Comparison of tightly controlled dose reduction of biologics with usual care for patients with psoriasis: a randomized clinical trial. JAMA Dermatol.

[3] Baniandres O, Rodriguez-Soria VJ, Romero-Jimenez RM, Suarez R (2015). Dose modification in biologic therapy for moderate to severe psoriasis: a descriptive analysis in a clinical practice setting. Actas Dermosifiliogr.

[4] Blauvelt A, Ferris LK, Yamauchi PS, Qureshi A, Leonardi CL, Farahi K, Fakharzadeh S, Hsu MC, Li S, Chevrier M, Smith K, Goyal K, Chen Y, Munoz-Elias EJ, Callis Duffin K (2017). Extension of ustekinumab maintenance dosing interval in moderate-to-severe psoriasis: results of a phase IIIb, randomized, double-blinded, active-controlled, multicentre study (PSTELLAR). Br J Dermatol.

[5] Conrad C, Gilliet M (2018). Psoriasis: from pathogenesis to targeted therapies. Clin Rev Allergy Immunol.

[6] Daudén E, Puig L, Ferrándiz C, Sánchez-Carazo JL, Hernanz-Hermosa JM (2016). Consensus document on the evaluation and treatment of moderate-to-severe psoriasis: psoriasis Group of the Spanish Academy of Dermatology and Venereology. J Eur Acad Dermatol Venereol.

[7] Flottorp SA, Oxman AD, Krause J, Musila NR, Wensing M, Godycki-Cwirko M, Baker R, Eccles MP (2013). A checklist for identifying determinants of practice: a systematic review and synthesis of frameworks and taxonomies of factors that prevent or enable improvements in healthcare professional practice. Implement Sci.

[8] Hamadah IR, Al Raddadi AA, Bahamdan KA, Fatani MI, Alnahdi A, Al Rakban AM, Alkhalifah A, Al Ameer A, Shaikh YH, Elgendi AM, Al Zoman AY, Alafif KA (2015). Saudi practical guidelines on biologic treatment of psoriasis. J Dermatolog Treat.

[9] Hansel K, Bianchi L, Lanza F, Bini V, Stingeni L (2017). Adalimumab dose tapering in psoriasis: predictive factors for maintenance of complete clearance. Acta Derm Venereol.

[10] Lebwohl M, Rivera-Oyola R, Murrell DF (2020). Should biologics for psoriasis be interrupted in the era of COVID-19?. J Am Acad Dermatol.

[11] Mahil SK, Wilson N, Dand N, Reynolds NJ, Griffiths CEM, Emsley R, Marsden A, Evans I, Warren RB, Stocken D, Barker JN, Burden AD, Smith CH, group Bs, the Pc, (2020). Psoriasis treat to target: defining outcomes in psoriasis using data from a real-world, population-based cohort study (the British Association of Dermatologists Biologics and Immunomodulators Register, BADBIR). Br J Dermatol.

[12] Mahil SK, Dand N, Mason KJ, Yiu ZZN, Tsakok T, Meynell F, Coker B, McAteer H, Moorhead L, Mackenzie T, Rossi MT, Rivera R, Mahe E, Carugno A, Magnano M, Rech G, Balogh EA, Feldman SR, De La Cruz C, Choon SE, Naldi L, Lambert J, Spuls P, Jullien D, Bachelez H, McMahon DE, Freeman EE, Gisondi P, Puig L, Warren RB, Di Meglio P, Langan SM, Capon F, Griffiths CEM, Barker JN, Smith CH, PsoProtect Study Group (2021). Factors associated with adverse COVID-19 outcomes in patients with psoriasis-insights from a global registry-based study. J Allergy Clin Immunol.

[13] Michielsens CAJ, van Muijen ME, Verhoef LM, van den Reek J, de Jong E (2021). Dose tapering of biologics in patients with psoriasis: a scoping review. Drugs.

[14] Mrowietz U, Kragballe K, Reich K, Spuls P, Griffiths CE, Nast A, Franke J, Antoniou C, Arenberger P, Balieva F, Bylaite M, Correia O, Dauden E, Gisondi P, Iversen L, Kemeny L, Lahfa M, Nijsten T, Rantanen T, Reich A, Rosenbach T, Segaert S, Smith C, Talme T, Volc-Platzer B, Yawalkar N (2011). Definition of treatment goals for moderate to severe psoriasis: a European consensus. Arch Dermatol Res.

[15] Ovejero-Benito MC, Munoz-Aceituno E, Sabador D, Reolid A, Llamas-Velasco M, Prieto-Perez R, Abad-Santos F, Dauden E (2020). Polymorphisms associated with optimization of biological therapy through drug dose reduction in moderate-to-severe psoriasis. J Eur Acad Dermatol Venereol.

[16] Piaserico S, Gisondi P, De Simone C, Marinello E, Conti A, Amerio P, Peserico A (2016). Down-titration of adalimumab and etanercept in psoriatic patients: a multicentre observational study. Acta Derm Venereol.

[17] Puig L, Carrascosa JM, Carretero G, de la Cueva P, Lafuente-Urrez RF, Belinchón I, Sánchez-Regaña M, García-Bustínduy M, Ribera M, Alsina M, Ferrándiz C, Fonseca E, García-Patos V, Herrera E, López-Estebaranz JL, Marrón SE, Moreno JC, Notario J, Rivera R, Rodriguez-Cerdeira C, Romero A, Ruiz-Villaverde R, Taberner R, Vidal D (2013). Spanish evidence-based guidelines on the treatment of psoriasis with biologic agents, 2013. Part 1: on efficacy and choice of treatment. Spanish Psoriasis Group of the Spanish Academy of Dermatology and Venereology. Actas Dermosifiliogr.

[18] Reich K, Puig L, Szepietowski JC, Paul C, Lacour JP, Tsianakas A, Sieder C, Rissler M, Pournara E, Orsenigo R (2019). Secukinumab dosing optimization in patients with moderate to severe plaque psoriasis: results from the randomised, open-label OPTIMISE study. Br J Dermatol.

[19] Romero-Jimenez RM, Escudero-Vilaplana V, Baniandres Rodriguez O, Garcia Martin E, Mateos Mayo A, Sanjurjo Saez M (2018). Association between clinical factors and dose modification strategies in the treatment with ustekinumab for moderate-to-severe plaque psoriasis. J Dermatolog Treat.

[20] Taniguchi T, Noda S, Takahashi N, Yoshimura H, Mizuno K, Adachi M (2013). An observational, prospective study of monthly adalimumab therapy for disease maintenance in psoriasis patients: a possible new therapeutic option for good responders to the initial induction treatment. J Eur Acad Dermatol Venereol.

[21] van Bezooijen JS, van Doorn MBA, Schreurs MWJ, Koch BCP, Te Velthuis H, Prens EP, van Gelder T (2017). Prolongation of biologic dosing intervals in patients with stable psoriasis: a feasibility study. Ther Drug Monit.

[22] van Muijen ME, van der Schoot LS, Bovenschen HJ, Dodemont SRP, van Lümig PPM, van Enst WA, van den Reek JMPA, de Jong EMGJ (2021). Dosisvermindering van biologics voor psoriasis. Ned Tijdschr Dermatol Venereol.

