# Supplementary material for: Attitudes and behaviour regarding dose reduction of biologics for psoriasis: a survey among dermatologists worldwide
Source: Arch Dermatol Res. 2021 Aug 31;314(7):687–95. doi: 10.1007/s00403-021-02273-4 (PMC9307528; doi:10.1007/s00403-021-02273-4)
Supplement: Supplementary file 1 — (PDF 409 KB) [file 403_2021_2273_MOESM1_ESM.pdf]

Supplementary Information

Manuscript title: Attitudes and behaviour regarding dose reduction of biologics for psoriasis: a survey among dermatologists worldwide.

Authors:

M.E. van Muijen,[1,2*] L.S. van der Schoot,[1,2*] J.M.P.A. van den Reek,[1,2] E.M.G.J. de Jong[1,2,3]

Affiliated institutions:

[1] Radboud University Medical Center (Radboudumc), Department of Dermatology, Nijmegen, the Netherlands

[2] Radboud University Medical Center (Radboudumc), Radboud Institute for Health Sciences (RIHS), Nijmegen, the Netherlands.

[3] Radboud University, Nijmegen, the Netherlands.

Corresponding author:

L.S. van der Schoot

René Descartesdreef 1

PO Box 9101

6500 HB Nijmegen, The Netherlands

E-mail address: lara.vanderschoot@radboudumc.nl

Content

Supplement S1. IPC Survey on dose reduction of biologic therapies in psoriasis.................................. 2

Table S1. Detailed answers per respondent to the question 'When do you consider dose reduction, regarding disease severity and safety?' 10

Table S2. Detailed answers per respondent to the questions 'How long do you treat a patient with a biological before you consider dose reduction?' and 'How long should your patient have stable low disease activity before you apply dose reduction?' 12

Table S3. Detailed answers per respondent to the question 'When do you stop dose reduction and decide to re-increase the dose in general?' 14

Supplement S1. IPC Survey on dose reduction of biologic therapies in psoriasis

Dear Sir/Madam,

We would like to get insight in thoughts and behaviour regarding dose tapering of biologics for psoriasis by dermatologists worldwide.
We would kindly ask you to fill in our questionnaire on this matter. Even if you do not apply dose reduction in daily practice, your answers are very much appreciated.
Completing the questionnaire will take approximately 15 minutes and will be anonymous.
In this questionnaire, the term 'dose reduction' can apply to both the prolongation of the dose intervals between two gifts, and lowering the dose of biologic per gift.

Thank you in advance.

We would kindly ask you to complete the following questions:

1. **In which country do you work?**

2. **In what type of clinic do you work?**
   - ☐ Academic hospital/University medical center
   - ☐ General hospital
   - ☐ Private hospital/clinic/practice
   - ☐ Other

3. **What is your job title?**
   - ☐ Dermatologist
   - ☐ Dermatologist in training
   - ☐ Other

4. **Do you prescribe biologicals for psoriasis?**
   - ☐ Yes
   - ☐ No
   - ➔ ***If no: end of questionnaire***

5. **Which biologicals do you prescribe? (multiple answers can be checked)**
   - ☐ Etanercept
   - ☐ Adalimumab
   - ☐ Infliximab
   - ☐ Certolizumab-pegol
   - ☐ Ustekinumab
   - ☐ Secukinumab
   - ☐ Ixekizumab
   - ☐ Brodalumab
   - ☐ Guselkumab
   - ☐ Risankizumab
   - ☐ Tildrakizumab
   - ☐ Other …..

6. **Do you prescribe biosimilars? (multiple answers can be checked)**
   - ☐ No
   - ☐ Yes: for adalimumab
   - ☐ Yes: for etanercept
   - ☐ Yes: for infliximab

7. **How many patients are approximately treated with biologicals in your department?**

☐ < 25
☐ 25-50
☐ 75-100
☐ 100-150
☐ 150-200
☐ 200-250
☐ 250-300
☐ 300-350
☐ 350-400
☐ 400-450
☐ 450-500
☐ >500
☐ I do not know

8. **Do you use a clinical score to measure disease activity? (multiple answers can be checked)**

☐ PASI (Psoriasis Area and Severity Index)
☐ BSA (Body Surface Area)
☐ PGA (Physician Global Assessment)
☐ No, I do not use a disease activity score.
☐ Other:

9. **Do you apply dose reduction or dose tapering?**

☐ Yes
☐ No

➔ ***If no: go to question 21.***

Display This Question: If 'Do you apply dose reduction or dose tapering?' Yes Is Selected.
NB. Questions regarding each biological are displayed if biological is selected in question 5 (Which biological do you prescribe?).

10. **We are interested in the way dose reduction is applied. Please indicate for every type of biological you prescribe if, and how you apply dose reduction. There are multiple possible answers.**

Etanercept
Recommended maintenance dose: 50 mg 1x/week or 25 mg 2x/week. Multiple answers can be checked.

☐ I do not apply dose reduction for etanercept.
☐ Etanercept 25 mg/week
☐ Etanercept 25 mg/10 days
☐ Etanercept 50 mg/10 days
☐ Etanercept 50 mg/2 weeks
☐ Etanercept 50 mg/3 weeks
☐ I apply longer dose intervals than previously mentioned
☐ Other:

Adalimumab
Recommended maintenance dose: 40 mg/14 days (or 40 mg/week; this dose is outside the scope of this survey). Multiple answers can be checked.

☐ I do not apply dose reduction for adalimumab.
☐ Adalimumab 40 mg/3 weeks
☐ Adalimumab 40 mg/4 weeks
☐ I apply longer dose intervals than previously mentioned.

☐ Other:

Infliximab

Recommended maintenance dose: 5 mg/kg every 8 weeks. Multiple answers can be checked.

! Prolonged dose intervals are not recommended for infliximab, as this may lead to formation of antibodies and possible risk of infusion reactions.

☐ I do not apply dose reduction for infliximab
☐ I reduce the dose to 3 mg/kg every 8 weeks
☐ Other:

Certolizumab-pegol

Recommended maintenance dose: 200 mg/14 days (or 400 mg/14 days: this dose is outside the scope of this survey). Multiple answers can be checked.

☐ I do not apply dose reduction for certolizumab-pegol
☐ Certolizumab pegol 200 mg/3 weeks
☐ Certolizumab pegol 200 mg/4 weeks
☐ I apply longer dose intervals than previously mentioned.
☐ Other:

Ustekinumab

Recommended maintenance dose: 45 mg/12 weeks or 90 mg/12 weeks (this dose is covered in the next question). Multiple answers can be checked.

Maintenance dose of 45 mg/12 weeks:

☐ I do not prescribe ustekinumab 45 mg
☐ I do not apply dose reduction for 45 mg/12 weeks
☐ Ustekinumab 45 mg/13 weeks
☐ Ustekinumab 45 mg/14 weeks
☐ Ustekinumab 45 mg/15 weeks
☐ Ustekinumab 45 mg/16 weeks
☐ Ustekinumab 45 mg/17 weeks
☐ Ustekinumab 45 mg/18 weeks
☐ Ustekinumab 45 mg/19 weeks
☐ Ustekinumab 45 mg/20 weeks
☐ Ustekinumab 45 mg/21 weeks
☐ Ustekinumab 45 mg/22 weeks
☐ Ustekinumab 45 mg/23 weeks
☐ Ustekinumab 45 mg/24 weeks
☐ I apply longer dose intervals than previously mentioned.
☐ Other:

Maintenance dose of 90 mg/12 weeks:

☐ I do not prescribe ustekinumab 90 mg.
☐ I do not apply dose reduction for ustekinumab 90 mg/12 weeks
☐ Ustekinumab 90 mg/13 weeks
☐ Ustekinumab 90 mg/14 weeks
☐ Ustekinumab 90 mg/15 weeks
☐ Ustekinumab 90 mg/16 weeks
☐ Ustekinumab 90 mg/17 weeks
☐ Ustekinumab 90 mg/18 weeks
☐ Ustekinumab 90 mg/19 weeks
☐ Ustekinumab 90 mg/20 weeks
☐ Ustekinumab 90 mg/21 weeks
☐ Ustekinumab 90 mg/22 weeks
☐ Ustekinumab 90 mg/23 weeks
☐ Ustekinumab 90 mg/24 weeks

- ☐ I reduce the dose of patients with stable low disease activity on 90 mg/12 weeks to 45 mg/12 weeks.
- ☐ I apply longer dose intervals than previously mentioned.
- ☐ Other:

<u>Secukinumab</u>
Recommended maintenance dose: 300 mg (2x150 mg)/4 weeks. Multiple answers can be checked.

- ☐ I do not apply dose reduction for secukinumab
- ☐ Secukinumab 300 mg/5 weeks
- ☐ Secukinumab 300 mg/6 weeks
- ☐ Secukinumab 300 mg/7 weeks
- ☐ Secukinumab 300 mg/8 weeks
- ☐ I reduce the dose to 150 mg/4 weeks
- ☐ I apply longer dose intervals than previously mentioned.
- ☐ Other:

<u>Ixekizumab</u>
Recommended maintenance dose: 80 mg/4 weeks. Multiple answers can be checked.

- ☐ I do not apply dose reduction for ixekizumab
- ☐ Ixekizumab 80 mg/5 weeks
- ☐ Ixekizumab 80 mg/6 weeks
- ☐ Ixekizumab 80 mg/7 weeks
- ☐ Ixekizumab 80 mg/8 weeks
- ☐ I apply longer dose intervals than previously mentioned.
- ☐ Other:

<u>Brodalumab</u>
Recommended maintenance dose: 210 mg/14 days. Multiple answers can be checked.

- ☐ I do not apply dose reduction for brodalumab
- ☐ Brodalumab 210 mg/3 weeks
- ☐ Brodalumab 210 mg/4 weeks
- ☐ I apply longer dose intervals than previously mentioned.
- ☐ Other:

<u>Guselkumab</u>
Recommended maintenance dose: 100 mg/8 weeks. Multiple answers can be checked.

- ☐ I do not apply dose reduction for guselkumab
- ☐ Guselkumab 100 mg/9 weeks
- ☐ Guselkumab 100 mg/10 weeks
- ☐ Guselkumab 100 mg/11 weeks
- ☐ Guselkumab 100 mg/12 weeks
- ☐ Guselkumab 100 mg/13 weeks
- ☐ Guselkumab 100 mg/14 weeks
- ☐ Guselkumab 100 mg/15 weeks
- ☐ Guselkumab 100 mg/16 weeks
- ☐ I apply longer dose intervals than previously mentioned.
- ☐ Other:

<u>Risankizumab</u>
Recommended maintenance dose: 150 mg (or 2x 75 mg)/12 weeks. Multiple answers can be checked.

- ☐ I do not apply dose reduction for risankizumab
- ☐ Risankizumab 150 mg/13 weeks
- ☐ Risankizumab 150 mg/14 weeks
- ☐ Risankizumab 150 mg/15 weeks
- ☐ Risankizumab 150 mg/16 weeks

- ☐ Risankizumab 150 mg/17 weeks
- ☐ Risankizumab 150 mg/18 weeks
- ☐ Risankizumab 150 mg/19 weeks
- ☐ Risankizumab 150 mg/20 weeks
- ☐ Risankizumab 150 mg/21 weeks
- ☐ Risankizumab 150 mg/22 weeks
- ☐ Risankizumab 150 mg/23 weeks
- ☐ Risankizumab 150 mg/24 weeks
- ☐ I reduce the dose to 75 mg/12 weeks
- ☐ I apply longer dose intervals than previously mentioned.
- ☐ Other:

<u>Tildrakizumab</u>

Recommended dose: 100 mg/12 weeks (or 200 mg/12 weeks; this dose is covered in the next question). Multiple answers can be checked.

<u>Maintenance dose of 100 mg/12 weeks:</u>

- ☐ I do not prescribe tildrakizumab 100 mg
- ☐ I do not apply dose reduction for tildrakizumab 100 mg/12 weken
- ☐ Tildrakizumab 100 mg/13 weeks
- ☐ Tildrakizumab 100 mg/14 weeks
- ☐ Tildrakizumab 100 mg/15 weeks
- ☐ Tildrakizumab 100 mg/16 weeks
- ☐ Tildrakizumab 100 mg/17 weeks
- ☐ Tildrakizumab 100 mg/18 weeks
- ☐ Tildrakizumab 100 mg/19 weeks
- ☐ Tildrakizumab 100 mg/20 weeks
- ☐ Tildrakizumab 100 mg/21 weeks
- ☐ Tildrakizumab 100 mg/22 weeks
- ☐ Tildrakizumab 100 mg/23 weeks
- ☐ Tildrakizumab 100 mg/24 weeks
- ☐ I apply longer dose intervals than previously mentioned.
- ☐ Other:

<u>Tildrakizumab</u>

Recommended dose: 100 mg/12 weeks (this dose was covered in the previous question) or 200 mg/12 weeks. Multiple answers can be checked.

<u>Maintenance dose of 200 mg/12 weeks:</u>

- ☐ I do not prescribe tildrakizumab 200 mg.
- ☐ I do not apply dose reduction for tildrakizumab 200 mg/12 weeks.
- ☐ Tildrakizumab 200 mg/13 weeks
- ☐ Tildrakizumab 200 mg/14 weeks
- ☐ Tildrakizumab 200 mg/15 weeks
- ☐ Tildrakizumab 200 mg/16 weeks
- ☐ Tildrakizumab 200 mg/17 weeks
- ☐ Tildrakizumab 200 mg/18 weeks
- ☐ Tildrakizumab 200 mg/19 weeks
- ☐ Tildrakizumab 200 mg/20 weeks
- ☐ Tildrakizumab 200 mg/21 weeks
- ☐ Tildrakizumab 200 mg/22 weeks
- ☐ Tildrakizumab 200 mg/23 weeks
- ☐ Tildrakizumab 200 mg/24 weeks
- ☐ I reduce the dose of patients with stable low disease activity on 200 mg/12 weeks to 100 mg/12 weeks.
- ☐ I apply longer dose intervals than previously mentioned.

☐ Other:

11. **How long do you treat a patient with a biological before you consider dose reduction?**

☐ After biological treatment for at least 3 months.
☐ After biological treatment for at least 6 months.
☐ After biological treatment for at least 9 months.
☐ After biological treatment for at least one year.
☐ My decision to apply dose reduction does not depend on treatment duration.
☐ Other:

12. **How long should your patients have stable low disease activity before you apply dose reduction?**

☐ I do not take the duration of stable low disease activity into account.
☐ Stable low diseae activity for at least 6 weeks
☐ Stable low diseae activity for at least 3 months
☐ Stable low diseae activity for at least 6 months
☐ Stable low diseae activity for at least 9 months
☐ Stable low disease activity for at least 1 year
☐ Other:

13. **When do you consider dose reduction, regarding disease severity and safety? (multiple answers can be checked)**

☐ When a patient has reached PASI (Psoriasis Area and Severity Index) 0 / is free from psoriasis
☐ When a patient has reached PASI ≤1
☐ When a patient has reached PASI ≤2
☐ When a patient has reached PASI ≤3
☐ When a patient has reached PASI ≤4
☐ When a patient has reached PASI ≤5
☐ When a patient has reached a BSA (Body Surface Area) ≤ ………….%
☐ When a patient has reached a PGA (Physician Global Assessment) of …………………..
☐ I make an estimation of the disease activity; when the psoriasis has (almost) cleared I consider dose reduction.
☐ If the patient experiences side effects.
☐ At patient's request.
☐ Other:

14. **In how many percent of your patients do you consider dose reduction approximately?** Please make an estimation.

☐ Never
☐ < 5 %
☐ 25-50 %
☐ 50-75%
☐ > 75 %

15. **Do you change the frequency of outpatient clinic visits after applying dose reduction in a patient? (multiple answers can be checked)**

☐ No
☐ Yes, extra telephone call
☐ Yes, extra outpatient clinic visit
☐ Yes, but I prolong the time between outpatient clinic visits.
☐ Only at patient's request.

☐ Other:

16. **When do you stop dose reduction and decide to re-increase the dose in general? (multiple answers can be checked)**

☐ When PASI increases >1
☐ When PASI increases >2
☐ When PASI increases >3
☐ When PASI increases >4
☐ When PASI increases >5
☐ When BSA increases > …………%
☐ When PGA increases > ………..
☐ When I consider the psoriasis severity to be ‘moderate’ disease.
☐ At patient’s request.
☐ Other:

17. **Could you indicate how often your patients agree to start dose tapering?**

☐ Never
☐ Rarely
☐ Sometimes
☐ Often
☐ Always

18. **Could you indicate in what percentage of your patients that underwent dose reduction, the dose reduction strategy was successful?** Please make an estimation.

☐ <20%
☐ 20-40%
☐ 40-60%
☐ 60-80%
☐ 80-100%
☐ I do not know

19. **What is/are your main reason(s) to apply dose reduction? (multiple answers can be checked)**

☐ Cost savings
☐ Safety / less side effects
☐ At patient’s request
☐ Other:

20. **Would you like to apply dose reduction more often than you do now?**

☐ Yes
☐ No

21. **Is/are there any reason(s) why you do not apply dose reduction as often as you would like to? (multiple answers can be checked)**

☐ No
☐ Yes, I don’t have enough time to discuss dose reduction with patients or guide them through the process
☐ Yes, I have not enough staff for support.
☐ Yes, I have only limited experience with dose reduction of biologics.
☐ Yes, I fear to apply dose reduction with biologics of the newer biologics (IL17/IL23).
☐ Yes, I often forget to discuss dose reduction with my patients.
☐ Yes, I fear financial consequences when I apply dose reduction more often.
☐ Yes, I often think patients do not want dose reduction.

☐ Other:

22. **Did you apply dose reduction more often during the COVID19 pandemic?**
    - ☐ Yes
    - ☐ No

Display This Question: If 'Do you apply dose reduction or dose tapering?' No Is Selected.

23. **What is/are the reason(s) for you not to apply dose reduction? (multiple answers can be checked)**
    - ☐ I think every patient needs the regular dose, as this is based on dose-finding studies.
    - ☐ There is no scientific proof that dose reduction is efficient/safe.
    - ☐ I do not want patients to have a psoriasis exacerbation because of dose reduction.
    - ☐ I have had bad experiences with dose reduction in the past.
    - ☐ I do not have time to apply dose reduction in daily practice (discuss with patients, treat possible exacerbations)
    - ☐ My experience with biologicals is too limited and therefore I prefer to prescribe the regular dose.
    - ☐ I do not know which way is the best to reduce the dose.
    - ☐ I fear antibody formation with a lower biological dose, and loss of efficacy.
    - ☐ I think patients do not want to reduce the dose due to fear of exacerbations.
    - ☐ I do not want to apply off-label dosing.
    - ☐ I have never considered dose reduction.
    - ☐ I think the costs of biologicals should be lowered instead of physicians applying dose reduction.
    - ☐ I fear financial consequences when I apply dose reduction. Patients who need a regular dose could be disadvantaged.
    - ☐ Other:

24. **Would you consider dose reduction if there was scientific evidence?**
    - ☐ Yes
    - ☐ No
    - ☐ Maybe

25. **What is your attitude towards dose reduction of biologicals for psoriasis?**
    - ☐ Positive
    - ☐ Neutral
    - ☐ Negative

26. **Do you feel the necessity for a guideline on how to apply dose reduction for biologicals?**
    - ☐ Yes
    - ☐ No
    - ☐ Other:

Display This Question: If 'Do you feel the necessity for a guideline on how to apply dose reduction for biologicals?' Yes Is Selected.

27. **What subjects should be covered in such a guideline? (multiple answers can be checked)**
    - ☐ Scientific background
    - ☐ Practical issues (time investment, eventual cost savings)
    - ☐ How to guide patients who start dose tapering.
    - ☐ Other:

Table S1. Detailed answers per respondent to the question 'When do you consider dose reduction, regarding disease severity and safety?'

	When do you consider dose reduction, regarding disease severity and safety? (multiple answers possible)														
**Respondent**	**PASI 0 / BSA 0 / PGA 0**	**PASI ≤ 1**	**PASI ≤ 2**	**PASI ≤ 3**	**PASI ≤ 4**	**PASI ≤ 5**	**BSA ≤ 1**	**BSA ≤ 2**	**BSA ≤ 3**	**BSA ≤ 5**	**PGA ≤ 1**	**General estimation of low disease activity**	**In case of side effects**	**At patients' request**	**Other**
1	x														
2												x		x	
3		x													
4	x														
5										x					
6	x	x										x	x	x	
7															x
8	x							x				x			
9	x														
10	x						x					x			
11	x												x	x	
12											x			x	
13			x					x			x	x			
14	x														
15		x	x										x		
16		x										x	x		
17	x														
18	x	x					x				x	x			
19												x			
20	x														
21			x												
22				x											
23									x						
24	x	x	x					x			x				
25		x					x				x	x	x		
26	x	x										x		x	
27	x	x					x					x		x	
28	x								x		x				
29	x	x											x		
30	x	x	x											x	
31												x		x	x
32	x	x	x	x	x	x									
33	x														

34												x			
35	x														
36				x			x				x		x	x	
37			x					x						x	
**Total**	**20**	**12**	**7**	**3**	**1**	**1**	**5**	**4**	**2**	**1**	**7**	**13**	**7**	**10**	**2**

Table S2. Detailed answers per respondent to the questions 'How long do you treat a patient with a biological before you consider dose reduction?' and 'How long should your patient have stable low disease activity before you apply dose reduction?'

	How long do you treat a patient with a biological before you consider dose reduction?[a]						How long should your patient have stable low disease activity before you apply dose reduction?[c]						
Respondent	≥ 3 months	≥ 6 months	≥ 1 year	≥ 2 years[b]	Does not depend on treatment duration	Missing	≥ 6 weeks	≥ 3 months	≥ 6 months	≥ 9 months	≥ 1 year	≥ 2 years[b]	Missing
1			x										x
2		x					x						
3			x								x		
4			x								x		
5					x			x					
6			x						x				
7			x								x		
8		x						x					
9		x						x					
10			x								x		
11			x								x		
12					x			x					
13			x								x		
14	x							x					
15			x								x		
16			x						x				
17			x								x		
18		x						x					
19			x										x
20			x						x				
21			x								x		
22			x						x				
23					x				x				
24			x								x		
25		x							x				
26			x							x			
27			x						x				
28		x						x					
29			x								x		
30			x						x				
31						x					x		

32		x						x					
33			x								x		
34				x								x	
35			x								x		
36			x								x		
37			x							x			
**Total**	**1**	**7**	**24**	**1**	**3**	**1**	**1**	**8**	**8**	**2**	**15**	**1**	**2**

[a] '≥ 9 months' was a fixed answer option to this question, but was not selected by any of the respondents.
[b] '≥ 2 years' was not a fixed answer option, but was answered in the comment section.
[c] 'I do not take the duration of stable low disease activity into account' was a fixed answer option to this question, but was not selected by any of the respondents.

Table S3. Detailed answers per respondent to the question 'When do you stop dose reduction and decide to re-increase the dose in general?'

	When do you stop dose reduction and decide to re-increase the dose in general? (multiple possible answers)														
Respondent	PASI ≥ 2	PASI ≥ 3	PASI ≥ 5	BSA ≥ 0%	BSA ≥ 2 %	BSA ≥ 3 %	BSA ≥ 5 %	BSA ≥ 7 %	BSA ≥ 10 %	PGA ≥ 0	PGA ≥ 1	PGA ≥ 2	Estimation 'moderate disease'	At patients' request	Other
1		x													
2															x
3		x													
4															x
5									x				x	x	
6		x												x	x
7															x
8								x					x		
9			x												
10		x					x						x		
11															x
12												x		x	
13	x				x						x		x		
14													x	x	
15		x													
16		x													
17															x
18													x		
19						x								x	
20				x						x					
21		x													
22			x												
23							x								x
24		x				x					x				
25			x										x		
26															x
27		x				x							x	x	
28												x	x		
29		x													
30		x													
31														x	x
32			x										x	x	
33			x												
34													x		

35													x		
36			x			x						x	x	x	
37		x													
**Total**	**1**	**12**	**6**	**1**	**1**	**4**	**2**	**1**	**1**	**1**	**2**	**3**	**13**	**9**	**9**
